# Beyond Restriction: Relationship Quality and Gender as Moderators of the Association Between Parental Restrictive Mediation and Adolescent Cyberbullying in China

**DOI:** 10.3390/children12121604

**Published:** 2025-11-25

**Authors:** Xiaolong Xie, Bowen Xiao, Yihao Hu, Jennifer Shapka, Junsheng Liu

**Affiliations:** 1 School of Psychology and Cognitive Science, East China Normal University, No. 3663, North Zhongshan Road, Shanghai 200062, China; xie.x.long@163.com (X.X.); hyh_psyworld@163.com (Y.H.); 2 College of Luxun Humanities, Shaoxing University, No. 1077, Chengnan Road, Shaoxing 312000, China; 3 Department of Educational and Counselling Psychology, and Special Education, University of British Columbia, 2125 Main Mall, Vancouver, BC V6T 1Z4, Canada; bowen.xiao@ubc.ca (B.X.); jennifer.shapka@ubc.ca (J.S.)

**Keywords:** parental restrictive mediation, parent–child relationship, cyber-aggression, cyber-victimization, gender, Chinese adolescents

## Abstract

**Highlights:**

**Main findings:**

- This study reveals a positive association between parental restrictive mediation and adolescent cyberbullying (including both aggression and victimization) in the Chinese context, challenging the conventional perception of restrictive mediation as a protective factor.
- Parent–child alienation is identified as a critical moderator. Specifically, under conditions of high alienation, restrictive mediation predicts increased cyberbullying involvement, with this exacerbating effect being particularly pronounced among adolescent boys.

**Implications of the main findings:**

- These findings provide a pivotal explanation for the contradictory evidence in the field, underscoring that parent–child relationship quality is a fundamental prerequisite for assessing the efficacy of parenting practices.
- The results strongly suggest that effective cyberbullying prevention must pivot from mere technical oversight to fostering trusting and emotionally connected parent–child relationships—a transition of particular importance for interventions targeting adolescent boys.

**Abstract:**

**Background/Objectives**: Although parental mediation has been widely recognized as a protective factor against cyberbullying, evidence regarding restrictive mediation remains inconsistent and sometimes contradictory. This inconsistency underscores the need to identify potential moderators. The present study examines whether parent–child relationship qualities (trust and alienation) and child gender moderate the associations between restrictive mediation and both cyber-aggression and cyber-victimization. **Methods**: Participants included 2075 adolescents (*M_age_* = 16.50, *SD* = 3.11; 926 boys) from Grades 7–12 in urban China. Self-report measures assessed restrictive mediation, parent–child trust and alienation, and adolescents’ involvement in cyber-aggression and cyber-victimization. **Results**: Parental restrictive mediation and parent–child alienation were positively associated with adolescents’ cyber-aggression and cyber-victimization, whereas parent–child trust was negatively associated with both outcomes. Moreover, parent–child alienation significantly moderated the associations between parental restrictive mediation and cyberbullying, such that restrictive mediation predicted higher levels of cyber-aggression and cyber-victimization under conditions of greater alienation. These moderating effects were particularly pronounced among boys, whereas for girls, the association was weaker or non-significant. **Conclusions**: These findings suggest that inconsistencies in prior research may be explained by variations in parent–child alienation and gender. The results highlight the need to foster trust and reduce alienation in parent–child relationships, rather than relying solely on restrictive Internet control, to prevent adolescents’ involvement in cyberbullying.

## 1. Introduction

Cyberbullying has emerged as a major threat to adolescent mental health, given its high prevalence and severe psychological consequences amid adolescents’ growing internet use [1]. Parental restrictive mediation has been considered a potential protective factor against both cyber-aggression and cyber-victimization [2,3]. However, empirical findings remain inconsistent, with some studies suggesting protective effects while others report null or even risk-enhancing associations [4,5]. To clarify these discrepancies, the present study investigates whether parent–child relationship qualities—specifically trust and alienation—moderate the associations between parental restrictive mediation and cyberbullying behaviors among Chinese adolescents. We further hypothesize that these moderating effects vary by gender, given cultural differences in parental control and gendered expectations in Chinese families. This study aims to inform cyberbullying prevention in China and advance theoretical understanding of how family dynamics shape adolescents’ online behaviors.

### 1.1. Internet Use and Cyberbullying Among Adolescents

The integration of the Internet into adolescent life is now virtually ubiquitous, a trend observed on a global scale. In the United States, 95% of teens (approximately 24.74 million) use social media and have a smartphone, and nearly half say they are online almost constantly, according to a new Pew Research Center survey of U.S. teens ages 13 to 17 conducted in 2024 [6]. This pattern is mirrored, and even intensified, in China. By 2022, China had 193 million underage Internet users, with a penetration rate of 97.2%, but the rates for adolescents exceeded 99% [7]. This indicates that Internet usage is nearly universal among Chinese adolescents, and the absolute scale of this user group is far larger than the number of social media users in the same age cohort in the United States. Furthermore, adolescent internet users account for 14% of all Internet users in China [8], and the majority possess personal internet-connected devices [7], further underscoring their prominent presence and high level of accessibility in the digital ecosystem. However, such digital immersion is a double-edged sword. While it offers unprecedented avenues for learning, socialization, and identity exploration [9,10], it simultaneously exposes adolescents to significant perils. Among these, cyberbullying has been identified as one of the most prevalent and psychologically damaging threats to adolescent well-being [11], positioning young people in a uniquely precarious position between opportunity and harm.

Cyberbullying is widely conceptualized as intentional, repeated aggression perpetrated through digital channels, with cyber-victimization denoting the experience of being targeted by such acts [12,13]. Reported prevalence rates in the global literature, however, show striking disparity, with estimates for perpetration and victimization ranging from 5% to 66% and 2% to 84%, respectively—a variation largely attributable to methodological and cultural differences [14]. Notably, amidst this wide variability, studies consistently identify a critical 2% to 7% of adolescents entrenched in severe and persistent patterns of involvement [15]. Within the Chinese context, cyberbullying is of pressing concern. National surveys have consistently revealed alarming rates. For instance, 27.6% of adolescents reported having experienced online safety incidents in the past six months, and 40.1% of adolescent internet users lack the initiative to counteract cyber violence [7]. The psychological consequences of involvement, whether as a perpetrator or victim, are severe, significantly elevating risks for internalizing disorders, suicidal ideation, and attempts [16]. Yet, despite a proliferation of research, the precise familial dynamics—specifically, how parenting practices interact with the quality of the parent–child relationship to predict cyberbullying involvement—remain inadequately elucidated [17,18,19].

### 1.2. The Relationship Between Parental Restrictive Mediation and Cyber-Aggression and Cyber-Victimization

Parents remain a cornerstone of protection against adolescent risk-taking, even amidst growing peer influence. A key factor of this influence is parental mediation—strategies to regulate and guide youth media use [20,21]. Scholarship typically categorizes these strategies into three types: restrictive (rule-setting), active (discursive guidance), and co-viewing (shared use without commentary) [22]. A robust body of evidence positions active mediation as the benchmark for effective digital socialization, as it mitigates risks while promoting opportunities by fostering critical thinking and autonomy [23,24,25]. This clear consensus on active mediation highlights the contentious and mixed evidence regarding the efficacy of restrictive mediation—a paradox that this study aims to unravel by examining key moderating conditions.

In practice, parental rule-setting remains a common strategy for regulating online behavior [25]. This is particularly evident in China, where cultural values emphasize parental responsibility for children’s development and academic success, often leading to strong parental control [26]. However, there is no clear consensus regarding the efficacy of restrictive mediation. Empirical evidence regarding the effectiveness of restrictive mediation in reducing cyberbullying remains limited and inconsistent [27]. Some studies have identified a negative association between restrictive mediation and cyber-victimization [28], whereas others suggest that restrictive strategies primarily increase parents’ awareness of their child’s cyberbullying experiences rather than reducing such behaviors [29]. Longitudinal findings further indicate that insufficient parental monitoring predicts higher levels of cyberbullying perpetration, whereas greater parental control reduces adolescents’ impulsivity and risk-taking, thereby lowering cyber-victimization rates [4,30]. In contrast, several studies report no significant relationship between restrictive mediation and cyberbullying [31,32], and some even document a positive association [3,33]. These discrepancies may arise because parents tend to impose stricter control when they perceive their children to be at risk, or because restrictive control implemented without warmth and support can have unintended negative consequences [33]. This profound inconsistency suggests that the effects of restrictive mediation are not universal but are likely contingent on contextual and perceptual factors (e.g., supportive family environments) [27].

According to the traditional perspective, social-cognitive domain theory [34] posits that adolescents’ perceptions of parental authority shape the legitimacy and, consequently, the effectiveness of parental rules. From a developmental perspective, older adolescents increasingly regard parental regulation of personal domains, such as media use, as intrusive and illegitimate [35]. When restrictive mediation is perceived as illegitimate, it may evoke psychological reactance, leading adolescents to engage more—rather than less—in the very behaviors parents seek to restrict [36]. Empirical studies support this view, showing that overly controlling mediation is often ineffective and may even generate boomerang effects [37,38].

Prior research has predominantly focused on Western cultural contexts [39]. However, it is noteworthy that parental mediation strategies exhibit significant differences between Eastern and Western cultural spheres [40]. At the macro-cultural level, the parenting practices of Chinese parents are deeply influenced by Confucianism [41]. Compared to the more child-centric and relatively permissive parenting philosophy prevalent in the West, traditional Chinese parent–child relationships are often more hierarchical, emphasizing the cultivation of self-control, diligence, and filial piety, thereby encouraging adherence to cultural norms of filial devotion [42,43]. Furthermore, contemporary Chinese parenting practices are also shaped by various other societal forces, such as rapid marketization, urbanization, and globalization [44]. At the micro-practical level, Chinese parenting philosophies place strong emphasis on the concept of “*guan jiao*” (training), characterized by a high degree of parental control [45]. Although studies in Western countries often suggest that parental intervention manifested through intrusion, pressure, and dominance can be psychologically detrimental to children, such practices yield different outcomes within East Asian family contexts [46]. Given that East Asian cultural norms strongly emphasize filial piety and collectivist orientation, parental control in this cultural environment is more likely to be perceived by children as an expression of care and warmth, rather than an infringement upon personal autonomy [42]. Taken together, these insights not only challenge the direct application of Western-based theories to the Chinese context but also underscore the critical need to investigate whether the pathways linking restrictive mediation to cyberbullying outcomes are universal or uniquely shaped by culturally specific norms such as filial piety.

### 1.3. Moderating Role of Parent–Child Relationship Qualities

We posit that parent–child relationship quality, particularly along the core dimensions of trust and alienation, serves as a critical moderator of the efficacy of restrictive mediation. Trust—the child’s belief in the parent’s reliability, understanding, and respect—is foundational to a secure attachment bond and a key component of the positive internal working models that arise from this attachment. According to attachment theory, these internal working models foster psychosocial adaptation and mitigate aggressive tendencies [47]. Parents who cultivate trust through emotional responsiveness and consistent support equip adolescents with the resilience to interpret parental guidance, including restrictions, as legitimate and caring [48,49]. Substantial empirical evidence aligns with this view: democratic family environments are linked to better emotional regulation and lower bullying involvement [19,50], while perceived parental support buffers against cyber-victimization [51].

Conversely, alienation—characterized by emotional distance, a lack of attention, and perceived rejection—undermines this foundation and fosters a context where parental control is met with suspicion [47]. Within a high-trust relationship, restrictive mediation is more likely to be perceived as an expression of concern, thereby mitigating psychological reactance and allowing its protective intent to be realized. In contrast, when the same restrictive strategies are imposed within an alienated relationship, they are more likely to be perceived as illegitimate and controlling, potentially provoking defiance and exacerbating negative outcomes [5,52,53].

We therefore argue that it is not merely the presence of rules, but the relational context in which they are embedded—specifically, the degree of trust versus alienation—that determines their impact. Attachment theory provides a coherent framework for this view [54,55], and empirical evidence corroborates that parental warmth and support buffer against cyber-aggression [56,57], whereas a lack of connection predicts its increase [52]. On this basis, we hypothesize that parent–child trust and alienation specifically moderate the association between restrictive mediation and cyberbullying. We expect that higher trust will enhance the protective function of mediation, whereas greater alienation will exacerbate its adverse effects.

### 1.4. The Present Study

**Figure 1:**
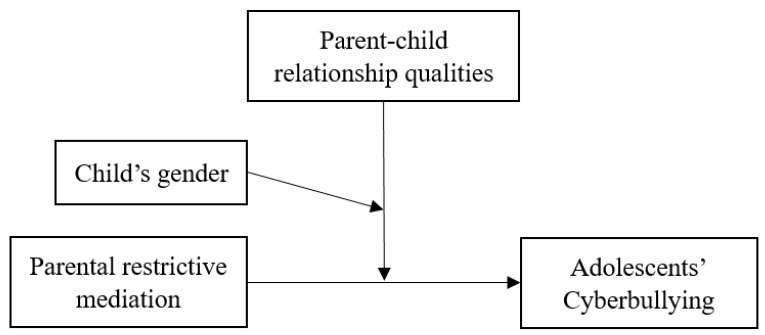
The hypothetical model of the study.

This study investigates the moderating role of parent–child relationship qualities in the association between parental restrictive mediation and cyberbullying among Chinese adolescents. Based on psychological reactance theory [36], we hypothesize that restrictive mediation will be positively associated with cyberbullying, especially when adolescents feel alienated from their parents, because such a context increases the likelihood that the mediation will be perceived as illegitimate control. In addition, given prior evidence that parental restrictive mediation strategies differ by child gender [3,58] and that cyberbullying involvement varies significantly between boys and girls [32], we further hypothesize that gender moderates the relationship between restrictive mediation and cyberbullying, with potentially distinct patterns for boys and girls within the Chinese cultural context. The hypothetical model of this study is shown in Figure 1. By examining how parent–child relationship quality and child gender interact with restrictive mediation, this study contributes to a more nuanced understanding of the pathways linking parental restrictive mediation to both cyber-aggression and cyber-victimization. These insights have practical implications for designing culturally sensitive parenting practices, educational programs, and policy interventions aimed at reducing online harm among adolescents.

## 2. Materials and Methods

### 2.1. Participants

The research participants were 2075 students (926 boys) from Grades 7 to 12 in four public middle schools in Fujian, China. Participants were recruited using cluster random sampling by selecting intact classes from Grades 7–12 across four public middle schools in Fujian Province. All students in the selected classes were invited to participate in the study. No specific exclusion criteria were applied; students not enrolled in the sampled classes were not included. The survey achieved a 100% completion rate with no missing data because the digital questionnaire required responses to all items, and participants complied with the procedure. Participants’ mean age was 16.50 years (SD = 3.11). All were of Han Chinese ethnicity (the dominant ethnic group in China), and 87% had one or more siblings, whereas 13% were the only child in their family. 99.1% of the households were intact families, while 0.9% were non-intact families. Most families were from middle to lower socioeconomic backgrounds. Regarding parental education, 68.4% of parents had completed junior high school, 20% high school, and 11.6% had a bachelor’s degree. 94.8% of parents were aware of their children’s use of internet media devices, while 5.2% reported being uninformed.

### 2.2. Procedure

The study was approved by the Institutional Review Board (IRB) of East China Normal University. Written informed consent was obtained from all participants and their parents prior to data collection. Data were collected by psychology graduate students from the East China Normal University during regular classroom sessions. Students completed questionnaires assessing parental mediation, parent–child attachment, and cyberbullying. Standardized instructions and clarifications were provided by the research team when necessary. To ensure linguistic equivalence, all measures were translated into Chinese and back-translated into English following established procedures.

### 2.3. Measures

#### 2.3.1. Parental Restrictive Mediation

Parental restrictive mediation was assessed using a 4-item scale adapted from the Parental Monitoring Scale (PMS) developed by Stattin and Kerr (2000) [59]. This scale measures the extent to which parents regulate their children’s online activities. A sample item is: “To what extent do you have to inform your parents about your planned online activities (e.g., what you will be doing, who you will be chatting with, or what content you will be posting)?” Participants responded on a 5-point Likert scale ranging from 1 (never) to 5 (all the time). The adapted version demonstrated good reliability and validity in Law, Shapka, and Olson (2010) [60]. In the present study, confirmatory factor analysis indicated excellent model fit: *χ*^2^*/df* = 2.93, CFI = 0.99, TLI = 0.99, RMSEA = 0.03, SRMR = 0.01. The internal consistency was good (Cronbach’s α = 0.79).

#### 2.3.2. Parent–Child Trust and Alienation

Adolescents’ perceptions of parent–child trust and alienation were assessed using the parent subscales of the Inventory of Parent and Peer Attachment—Short Form (IPPA-SF), developed by Raja, McGee, and Stanton (1992) [61]. This 12-item self-report measure evaluates three dimensions of parent–child attachment: Trust (4 items, e.g., “My father or mother respects my feelings”), Communication (4 items), and Alienation (4 items, e.g., “I don’t get much attention from my father or mother”). For the purposes of this study, only the Trust and Alienation subscales were utilized. All items were rated on a 5-point Likert scale ranging from 1 (almost never) to 5 (almost always). The IPPA has established good psychometric properties in Pan et al. (2020) [62]. In our sample, the internal consistency was good for the Trust subscale (α = 0.88) and the total score (α = 0.76), while the Alienation subscale demonstrated modest reliability (α = 0.61).

#### 2.3.3. Cyberbullying

Cyber-aggression and cyber-victimization were assessed using the Cyber-Aggression and Victimization Scale (CAV), developed by Shapka et al. (2018) [63]. The scale contains two distinct 6-item subscales measuring cyber-aggression (e.g., “Have you ever sent or forwarded a hurtful message electronically to someone”) and cyber-victimization (e.g., “Have you ever received a hurtful message from someone online”). All items were rated on a 5-point Likert scale ranging from 0 (never) to 4 (daily). The Chinese version of the CAV, validated by Xie et al. (2022) [64], has demonstrated strong psychometric properties. In the present study, the internal consistency was excellent for both the cyber-aggression (α = 0.91) and cyber-victimization (α = 0.90) subscales, as well as for the total score (α = 0.94).

### 2.4. Analytical Strategy

All analyses were performed in IBM SPSS (Version 20). Preliminary analyses examined correlations among the study variables. Hierarchical regression analyses were employed to test both main and moderating effects. For significant moderating effects, simple slope analyses were conducted following Aiken and West’s procedure [65]. These analyses examined the effects of parental restrictive mediation on cyberbullying at high and low levels of parent–child relationship quality (±1 *SD* from the mean).

## 3. Results

### 3.1. Preliminary Analyses

Table 1 displays descriptive statistics and zero-order correlations for all study variables. As shown in Table 1, the correlation analysis revealed that parental restrictive mediation was positively associated with both cyber-aggression (*r* = 0.11, *p* < 0.001) and cyber-victimization (*r* = 0.10, *p* < 0.001) and parent–child alienation was positively associated with both cyber-aggression (*r* = 0.24, *p* < 0.001) and cyber-victimization (*r* = 0.23, *p* < 0.001). In contrast, parent–child trust was negatively associated with cyber-aggression (*r* = −0.15, *p* < 0.001) and cyber-victimization (*r* = −0.16, *p* < 0.001). Regarding demographic variables, boys, compared to girls, reported significantly lower levels of parent–child trust (*r* = 0.04, *p* = 0.04), while reporting higher levels of both cyber-aggression (*r* = −0.14, *p* < 0.001) and cyber-victimization (*r* = −0.10, *p* < 0.001). Age was positively associated with parent–child alienation (*r* = 0.05, *p* = 0.03), cyber-aggression (*r* = 0.10, *p* < 0.001), and cyber-victimization (*r* = 0.08, *p* = 0.001). It should be noted that the internal consistency of the parent–child alienation subscale was modest (α = 0.61), which may have attenuated its observed associations with other variables.

### 3.2. Moderating Effects of Parent–Child Relationship Qualities on the Relationship Between Parental Restrictive Mediation and Cyber-Aggression

The analyses examined whether parent–child relationship qualities moderated the associations between parental restrictive mediation and cyberbullying, including cyber-aggression and cyber-victimization. A series of hierarchical regression models were conducted to test the hypothesized effects. In Step 1, gender and age were included as control variables. In Step 2, parental restrictive mediation was added, followed by one parent–child relationship quality (either trust or alienation) in Step 3. Step 4 included the two-way interactions between parental restrictive mediation, the moderator variables, and gender. Step 5 tested the three-way interactions. All continuous variables were standardized to reduce multicollinearity [65]. Results are summarized in Table 2 and Table 3.

**Figure 2:**
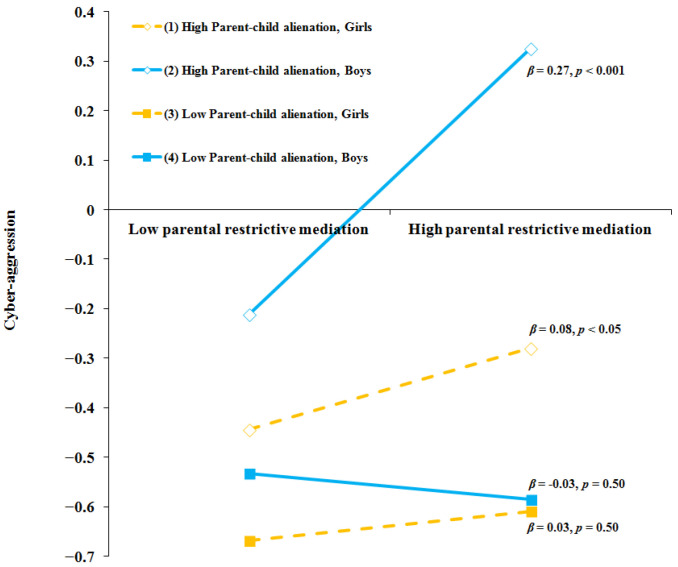
Moderating Effects of parent–child alienation and gender on relation between parental restrictive mediation and cyber-aggression.

As shown in Table 2 (cyber-aggression model), in predicting cyber-aggression, parental restrictive mediation (*β* = 0.13, *p* < 0.001) and parent–child alienation (*β* = 0.28, *p* < 0.001) showed significant positive main effects, whereas parent–child trust showed a significant negative main effect (*β* = −0.07, *p* = 0.028). A significant three-way interaction emerged between parental restrictive mediation, parent–child alienation, and gender (*β* = −0.12, *p* = 0.006). Simple slopes analysis showed that the association between restrictive mediation and cyber-aggression was stronger among boys with high parent–child alienation (*β* = 0.27, *p* < 0.001) than among girls (*β* = 0.08, *p* < 0.05). At low levels of parent–child alienation, the simple slopes for both boys and girls were non-significant. (see Figure 2).

### 3.3. Moderating Effects of Parent–Child Relationship Qualities on the Relationship Between Parental Restrictive Mediation and Cyber-Victimization

The analysis for cyber-victimization followed the same hierarchical regression procedures as detailed in Section 3.2. The results, summarized in Table 3, revealed a similar pattern of effects to those found for cyber-aggression.

**Figure 3:**
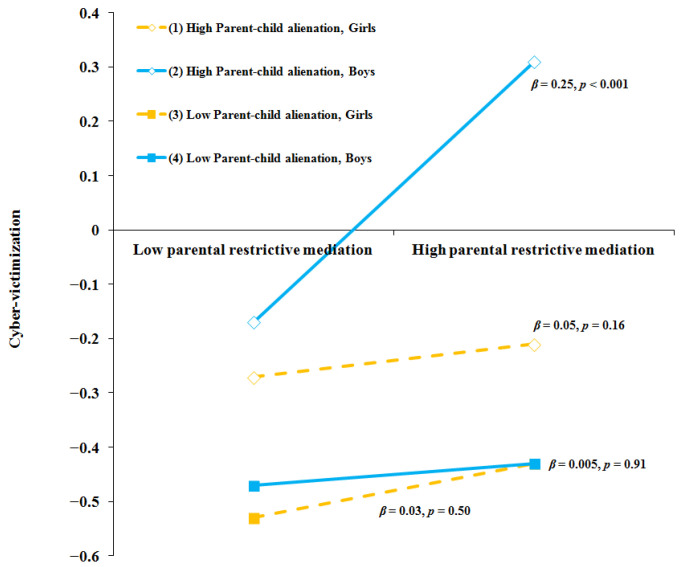
Moderating Effects of parent–child alienation and gender on relation between parental restrictive mediation and cyber-victimization.

In predicting cyber-victimization, parental restrictive mediation (*β* = 0.13, *p* < 0.001) and parent–child alienation (*β* = 0.26, *p* < 0.001) showed significant positive main effects, whereas parent–child trust showed a significant negative main effect (*β* = −0.07, *p* = 0.032). A significant three-way interaction emerged between parental restrictive mediation, parent–child alienation, and gender (*β* = −0.12, *p* = 0.005), indicating that the effect of restrictive mediation on cyber-victimization varied by both gender and levels of parent–child alienation. Simple slopes analysis indicated that parental restrictive mediation was positively associated with cyber-victimization only among boys with high levels of parent–child alienation (*β* = 0.25, *p* < 0.001), whereas the slopes for other groups were non-significant (see Figure 3).

## 4. Discussion

The proliferation of digital technology has posed significant challenges for parents, particularly in supervising adolescents’ online activities [27]. This study investigated how parent–child relationship qualities moderate the association between parental restrictive mediation and cyberbullying among Chinese adolescents. The findings were largely consistent with our hypotheses. Both parental restrictive mediation and parent–child alienation were significantly and positively associated with cyber-aggression and cyber-victimization. Notably, the moderation analyses revealed that the effects of parental restrictive mediation on cyberbullying were amplified among adolescents experiencing higher levels of parent–child alienation, a pattern that was particularly pronounced for boys.

### 4.1. Relationship Between Parental Restrictive Mediation and Cyberbullying

Our findings reveal that higher levels of parental restrictive mediation are associated with increased involvement in cyberbullying, both as perpetrators and victims. To explain this counterintuitive pattern, we draw on psychological reactance theory [36] as our central framework. This theory posits that individuals are motivated to protect their behavioral freedoms. When a freedom is threatened or eliminated—as adolescents may perceive with rigid parental restrictions—psychological reactance is aroused, often manifesting as defiance against the source of the threat [37,38].

In the context of our study, parental restrictions, though often well-intentioned, can be perceived by adolescents as intrusive and autonomy-threatening. This perception of a threatened freedom in their personal online domain is what triggers reactance. Here, social-cognitive domain theory [34] helps to clarify the antecedent of this perception—namely, why adolescents judge these parental restrictions as illegitimate intrusions into their personal domain. Psychological reactance theory [36], in turn, provides the mechanism for the subsequent behavioral escalation: the resulting defiant motivation can then directly drive oppositional behaviors, such as cyber-aggression, or lead to rash decisions that increase the risk of cyber-victimization. This pathway aligns with prior research documenting the counterproductive effects of coercive parental control [3,33,66].

Parental restrictions imposed without explanation or dialogue may undermine trust and motivate adolescents to assert autonomy by circumventing parental oversight. Such resistance often manifests in risky or oppositional online behaviors, thereby heightening the likelihood of both cyber-aggression and victimization. Moreover, restrictive mediation is frequently implemented without the complementary discussions that could equip adolescents with strategies to manage online risks [67]. Without such discussions, adolescents may lack the problem-solving skills necessary to navigate digital challenges, leaving them at heightened risk of negative online experiences [68].

Parental mediation of adolescents’ Internet use involves considerable complexities. On the one hand, adolescents’ developmental striving for autonomy reduces their receptiveness to direct parental restrictive mediation; on the other hand, the widespread use of mobile devices limits parents’ ability to provide continuous supervision [69]. These dynamics underscore the necessity for parents to achieve an appropriate balance between respecting adolescents’ autonomy and offering effective guidance to mitigate online risks.

### 4.2. The Moderating Role of Parent–Child Relationship Qualities and Adolescent Gender

As anticipated, parent–child trust was negatively associated with cyberbullying, underscoring its role as a protective factor. In contrast, parent–child alienation was positively related to greater involvement in both cyber-aggression and victimization. These results align with prior research, which has demonstrated that strong emotional bonds between parents and children reduce adolescents’ engagement in risky online behaviors [70,71]. Conversely, perceived alienation from parents appears to heighten the risk of cyberbullying involvement [51].

Our study contributes to the literature by providing evidence for the moderating role of parent–child relationship qualities in the link between parental restrictive mediation and cyberbullying. Specifically, the detrimental association between restrictive mediation and cyberbullying was significantly stronger for adolescents who perceived higher levels of alienation from their parents. This pattern suggests that alienation may intensify the detrimental effects of restrictive mediation, consistent with authoritarian parenting models characterized by rigid discipline and limited communication [72]. Such parenting practices, which often overlap with a context of high restrictive mediation, may collectively undermine adolescents’ need for autonomy, thereby increasing the likelihood of rebellious and risky behaviors, including cyberbullying [73].

Contrary to our hypothesis, parent–child trust did not significantly moderate the association between restrictive mediation and cyberbullying. This suggests that while trust is a robust standalone protective factor, it may not be sufficient to “neutralize” the potential iatrogenic effects of restrictive mediation. In other words, even in generally trusting relationships, adolescents may still perceive strict, non-negotiable rules as an infringement on their autonomy, leading to reactance. These findings indicate that trust serves as a fundamental protective factor, mitigating risk regardless of the degree of restrictive mediation. By contrast, alienation operates as a “risk amplifier”, especially under conditions of restrictive mediation.

Collectively, these findings underscore the importance of effective parent–child communication. It is crucial for parents to foster open and responsive dialogue while maintaining an appropriate balance between supervision and autonomy [55]. Furthermore, by providing structured opportunities for Internet use in conjunction with meaningful offline interactions, parents can better support adolescents in navigating online risks and reduce the likelihood of problematic behaviors such as cyberbullying.

Additionally, significant gender differences were observed. Boys were more likely to engage in cyber-aggression and victimization under conditions of restrictive mediation, particularly when parent–child alienation was high. While girls often appear to benefit from greater levels of parental mediation [28], boys’ typically lower levels of parent–child communication during adolescence may intensify the negative consequences of alienation. These patterns may reflect gendered parenting practices in Chinese families. Boys, who are often expected to be more independent, may react more strongly to interventions perceived as controlling. Conversely, heightened parental concerns about boys’ addiction or aggression can lead to stricter yet less effective regulation, thereby amplifying negative outcomes among boys. These findings suggest that gender-sensitive communication strategies could be particularly beneficial in addressing the unique vulnerabilities faced by boys [74].

### 4.3. Cultural Context and Theoretical Implications

Our findings regarding the exacerbating role of parent–child alienation and the pronounced effects among boys should be interpreted within the specific socio-cultural context of urban China. The Confucian tradition emphasizes hierarchical yet harmonious family relationships [41,42,43], where parental authority is often intertwined with expressions of care. In this framework, restrictive mediation from a parent in a high-alienation relationship may be perceived not merely as an inconvenience, but as a fundamental breach of the expected *en qing* (affection) that should undergird *guan jiao* (training). This violation of cultural scripts regarding appropriate parent–child interactions may intensify the psychological reactance experienced by adolescents, thereby amplifying counterproductive outcomes [53].

Furthermore, the stronger moderating effect observed among boys aligns with the distinct gender socialization practices prevalent in China [41]. It is plausible that boys are traditionally socialized to assert autonomy and embody strength, making them more sensitive to autonomy-threatening controls, especially within emotionally distant relationships. Their subsequent reactance may thus manifest more readily in externalizing behaviors like cyber-aggression. For girls, who may be socialized towards greater compliance, the pathway from control to overt aggression might be weaker or channeled into different behavioral outcomes [3,58].

These cultural nuances suggest that cyberbullying prevention programs in China must be culturally tailored. Moving beyond one-size-fits-all approaches, interventions should prioritize the cultivation of trust and the reduction in alienation as foundational steps. Efforts should help parents, particularly those of adolescent boys, to express concern and exercise guidance in ways that are perceived as legitimate and supportive, rather than solely restrictive, thereby mitigating the elicitation of defiant responses.

### 4.4. Limitations and Future Directions

While this study advances understanding of cyberbullying in non-Western societies, several methodological and contextual limitations should be acknowledged. First, the cross-sectional design limits the ability to infer causal relationships among parental mediation, parent–child relationship qualities, and cyberbullying. The relationships observed could be subject to bidirectional influences. For instance, while we hypothesized that parental restrictive mediation influences adolescent online behavior, it is equally plausible that adolescents’ experiences (e.g., being a victim of cyberbullying) could trigger more restrictive mediation as a protective measure by parents. Similarly, parents may implement restrictions in reaction to a child’s pre-existing behavioral problems related to their online activities at home or school. Future longitudinal research is essential to disentangle these complex temporal and causal pathways. Second, because the sample was drawn exclusively from urban areas in China, the findings may not be generalizable to rural populations or other cultural contexts. Future research should incorporate more diverse samples—including rural regions and varying socio-economic backgrounds—to assess the broader applicability of these results. Third, the reliance on adolescent self-reports via scales means that the data reflect subjective perceptions rather than objective relationship quality. We did not measure the dyadic construct of “parent–child relationship quality,” as the title may imply, but rather adolescents’ attachment and feelings within that relationship. The absence of parental data, behavioral observations, or clinical assessments limits the scope and depth of our conclusions. Future studies should include these multi-faceted measures. Fourth, while our study identified several statistically significant associations, it is important to note that the effect sizes were generally modest. This underscores that adolescent cyberbullying and cyber victimization are complex phenomena influenced by a multifaceted ecosystem of factors. The parent and child factors examined herein represent only a part of this puzzle; other elements, such as peer influences, school climate, and individual personality traits, are likely equally or more critical to a comprehensive understanding of these behaviors. Finally, the parent–child alienation subscale demonstrated only modest reliability (Cronbach’s α = 0.61), which may have attenuated its associations with other variables. Future studies should adopt more psychometrically robust measures to confirm its moderating role.

## 5. Conclusions

Our findings suggest that parental restrictive mediation may unintentionally contribute to cyberbullying, and this effect is amplified when adolescents experience alienation from their parents, particularly among boys. These results highlight the need for a balanced approach to parental mediation that supports adolescents’ autonomy while fostering responsible Internet use. Strengthening parent–child relationships—characterized by mutual trust and open dialogue—is essential for mitigating the adverse effects of restrictive mediation. Finally, collaborative efforts among parents, educators, and policymakers are required to develop strategies that harmonize adolescents’ online freedom with appropriate guidance and support, thereby reducing their vulnerability to cyberbullying.

## Figures and Tables

**Table 1:** Descriptive statistics and intercorrelations of study variables.

	1	2	3	4	5	6	7
1. Parental restrictive mediation	1						
2. Parent–child trust	0.001	1					
3. Parent–child alienation	0.09 ***	−0.43 ***	1				
4. Cyber-aggression	0.11 ***	−0.15 ***	0.24 ***	1			
5. Cyber-victimization	0.10 ***	−0.16 ***	0.23 ***	0.76 ***	1		
6. Gender	−0.04	0.04 *	0.03	−0.14 ***	−0.10 ***	1	
7. Age	−0.02	−0.03	0.05 *	0.10 **	0.08 **	−0.01	1
*M*	2.62	2.80	1.67	1.16	1.17	0.55	16.50
*SD*	10.51	0.65	0.61	0.39	0.40	0.50	3.11

Note: Gender: 0 = boys, 1 = girls; * *p* < 0.05, ** *p* < 0.01, *** *p* < 0.001.

**Table 2:** Moderating Effects of Parent–Child Relationship Qualities on Relation between Parental Restrictive Mediation and Cyber-aggression.

Predictor	Cyber-Aggression				
	*β*	*SE*	*t* Value	*p*	95% *CI*
Constant	−0.25	0.11	−2.169 *	0.033	[−0.47, −0.02]
Gender	−0.25	0.04	−5.957 ***	0.000	[−0.33, −0.16]
Age	0.02	0.01	3.358 **	0.001	[0.01,0.04]
Parental restrictive mediation	0.13	0.03	4.189 ***	0.000	[0.07, 0.19]
Parent–child trust	−0.07	0.03	−2.204 *	0.028	[−0.14, −0.09]
Parent–child alienation	0.28	0.03	8.418 ***	0.000	[0.21, 0.34]
Parental restrictive mediation × Gender	−0.07	0.04	−1.609	0.108	[−0.15, 0.02]
Parent–child trust × Gender	0.03	0.05	0.692	0.489	[−0.06, 0.12]
Parent–child alienation × Gender	−0.16	0.05	−3.485 ***	0.001	[−0.25, −0.07]
Parental restrictive mediation × Parent–child trust	−0.04	0.03	−1.390	0.165	[−0.10, 0.02]
Parental restrictive mediation × Parent–child alienation	0.13	0.03	4.390 ***	0.000	[0.07, 0.19]
Parental restrictive mediation × Parent–child trust × Gender	0.07	0.04	0.395	0.693	[−0.06, 0.10]
Parental restrictive mediation × Parent–child alienation × Gender	−0.12	0.04	−2.744 **	0.006	[−0.20, −0.03]

Note: All variables were standardized; Gender: 0 = boys, 1 = girls; * *p* < 0.05; ** *p* < 0.01; *** *p* < 0.001.

**Table 3:** Moderating Effects of Parent–Child Relationship Qualities on Relation between Parental Restrictive Mediation and Cyber-victimization.

Predictor	Cyber-Victimization				
	*β*	*SE*	*t* Value	*p*	95% *CI*
Constant	−0.19	0.11	−1.657	0.098	[−0.41, 0.04]
Gender	−0.17	0.04	−4.072 ***	0.000	[−0.25, −0.09]
Age	0.02	0.01	2.445 *	0.015	[0.003, 0.03]
Parental restrictive mediation	0.13	0.03	4.336 ***	0.000	[0.07, 0.19]
Parent–child trust	−0.07	0.03	−2.149 *	0.032	[−0.14, −0.01]
Parent–child alienation	0.26	0.04	7.724 ***	0.000	[0.19, 0.32]
Parental restrictive mediation × Gender	−0.09	0.05	−2.042 *	0.041	[−0.17, −0.003]
Parent–child trust × Gender	0.01	0.05	0.244	0.807	[−0.08, 0.10]
Parent–child alienation × Gender	−0.14	0.03	−3.082 **	0.002	[−0.23, −0.05]
Parental restrictive mediation × Parent–child trust	−0.02	0.03	−0.754	0.451	[−0.08, 0.04]
Parental restrictive mediation × Parent–child alienation	0.11	0.03	3.730 ***	0.000	[0.05, 0.17]
Parental restrictive mediation × Parent–child trust × Gender	−0.02	0.04	−0.506	0.613	[−0.10, 0.06]
Parental restrictive mediation × Parent–child alienation × Gender	−0.12	0.04	−2.805 **	0.005	[−0.20, −0.04]

Note: All variables were standardized; Gender: 0 = boys, 1 = girls; * *p* < 0.05; ** *p* < 0.01; *** *p* < 0.001.

## Data Availability

The data presented in this study are available on request from the corresponding author. The data are not publicly available due to ethical principles.
